# A case of successful catheter removal with ventricular septal defect occluder after accidental misplacement of left subclavian vein catheter into aortic arch

**DOI:** 10.1016/j.radcr.2025.12.062

**Published:** 2026-01-22

**Authors:** Chuke Qiao, Shaojie Liu, Hongwei Shan

**Affiliations:** a Department of Emergency Medicine, Affiliated Hospital of Inner Mongolia Medical University, No. 5, Tongdao North Street, Huimin District, Hohhot City, Inner Mongolia Autonomous Region, 010100, China; b Department of General Surgery, Affiliated Hospital of Inner Mongolia Medical University, No. 5, Tongdao North Street, Huimin District, Hohhot City, Inner Mongolia Autonomous Region, 010100, China

**Keywords:** Left subclavian vein catheterization, Postaortic catheterization, Ventricular septal defect occluder

## Abstract

Abstract The subclavian vein is one of the most frequently-used approaches for central venous catheterization. However, it demands high technical skills and accurate puncture positioning. In case of accidental misplacement into the artery, it is impossible to achieve sufficient local hemostasis. Moreover, removing the catheter without assistance may lead to severe complications or even death. This paper reports a case of an elderly patient with multiple underlying diseases. After left subclavian vein catheterization accidentally entered the aortic arch, a ventricular septal defect occluder was used to successfully remove the catheter. There were no adverse clinical sequelae after the operation, providing a new solution for the accidental insertion of a catheter into an artery in noncompressible areas.

## Introduction

Central venous catheterization (CVC) via the subclavian vein is widely used in critically ill patients for fluid resuscitation, vasoactive drug administration, hemodialysis, and parenteral nutrition. The subclavian approach offers advantages such as lower infection rates and better catheter stability compared with femoral access, but it is associated with a steeper learning curve and a higher risk of serious vascular complications due to its proximity to the subclavian artery and the noncompressible nature of the thoracic outlet. Accidental arterial puncture or catheter misplacement can result in life-threatening hemorrhage, pseudoaneurysm, arteriovenous fistula, or even death, particularly when the catheter is removed without adequate hemostasis. Management strategies for such complications are limited, especially when the catheter tip is positioned in a major intrathoracic artery that cannot be compressed externally. Herein, we report a case of inadvertent placement of a subclavian catheter into the aortic arch in a critically ill elderly patient with multiple comorbidities, which was successfully managed using a ventricular septal defect (VSD) occluder to facilitate safe catheter removal. This case highlights a novel and effective approach for the management of arterial misplacement in noncompressible regions, with potential implications for clinical practice.

## Case description

**Fig. 1:**
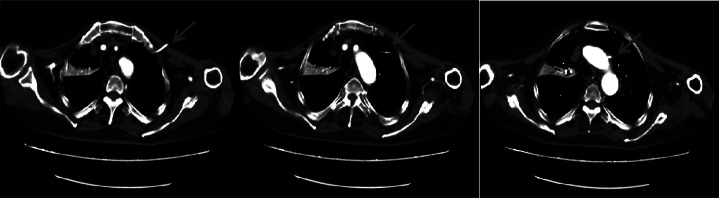
Chest CT scan showing accidental cannulation of the left subclavian central venous catheter into the aortic arch; the arrow indicates the aortic puncture site.

**Fig. 2:**
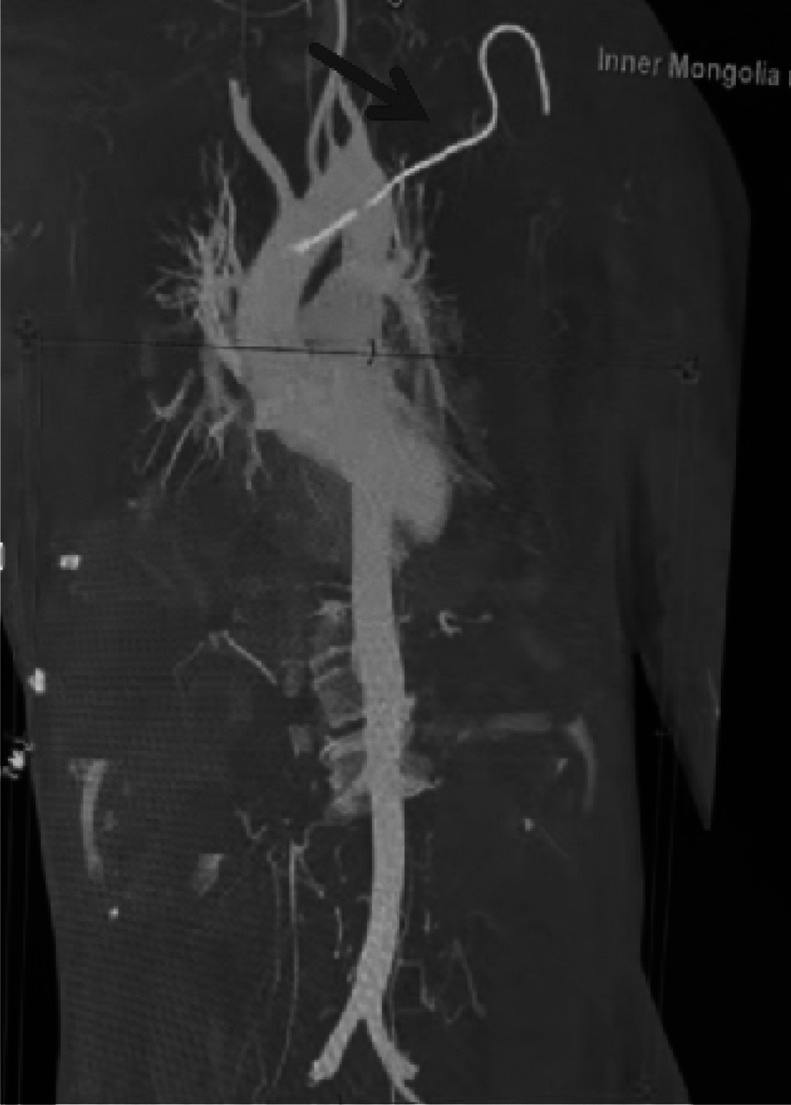
CT angiography of the entire aorta; the arrow indicates the aortic catheter placement.

A 65-year-old male patient presented to the Emergency Intensive Care Unit with ``intermittent convulsions for 2 months and fever for over 1 month''(Ethical approval is not applicable to this article). During hospitalization, a 6F left subclavian central venous catheter was inserted. Chest computed tomography (CT) (Fig. 1) revealed accidental cannulation of the aortic arch. Emergency CT angiography (CTA) of the entire aorta (Fig. 2) confirmed the postcatheterization changes in the aortic arch. Following comprehensive evaluation, a decision was made to attempt catheter removal and arterial puncture closure using a ventricular septal defect (VSD) occluder. After obtaining informed consent from the patient’s family, the patient was transferred to the cardiac catheterization laboratory.

**Fig. 3:**
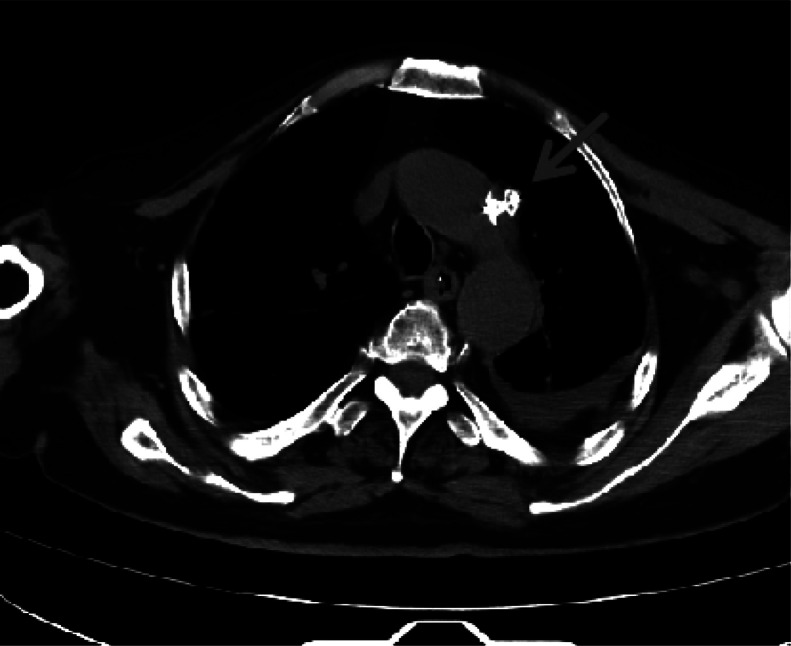
Aortic angiography after interventional catheter removal.

**Fig. 4:**
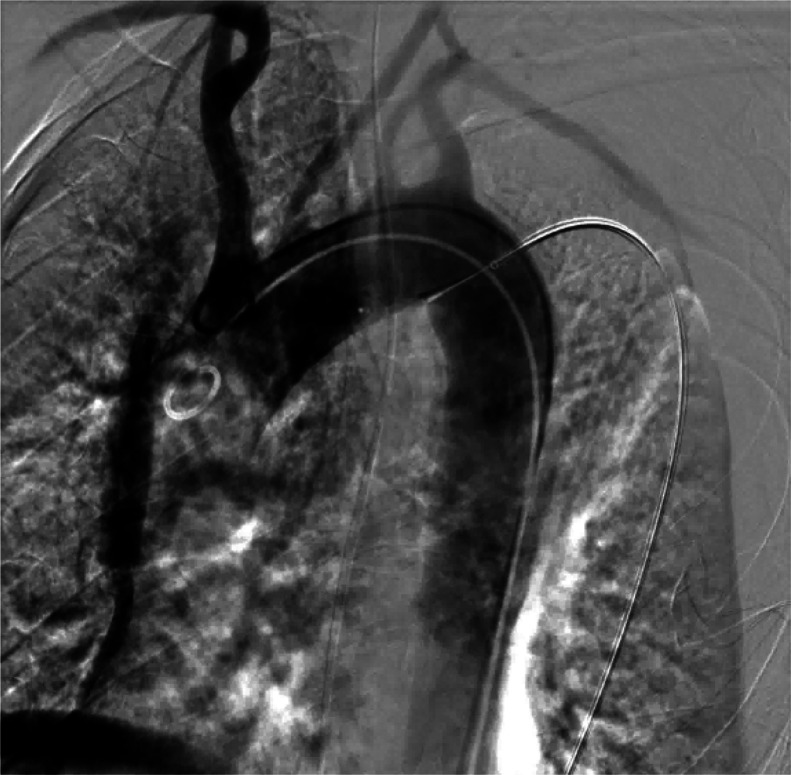
Postoperative chest CT re-examination; the arrow indicates the ventricular septal defect occluder.

Under aseptic conditions and local anesthesia, a 6F sheath was inserted via puncture of the right femoral artery. A hydrophilic guidewire was advanced through the aortic catheter to the aortic sinus, after which the misplaced catheter was withdrawn. The occluder delivery system was introduced along the hydrophilic guidewire. A basket guidewire was then inserted through the femoral artery sheath, snared the hydrophilic guidewire at the aortic sinus, and pulled it to the femoral artery exit for fixation. The occluder was delivered via its delivery system. A contrast catheter was advanced along the femoral artery guidewire to the aortic arch for angiography, which confirmed proper positioning of the occluder prior to release (Fig. 3). Repeat angiography demonstrated no leakage around the occluder. X-ray examination ruled out pneumothorax and pleural effusion. The occluder delivery system and contrast catheter were removed, and hemostasis at the femoral artery puncture site was achieved via manual compression. The aortic vessel occlusion procedure was successful (Fig. 4).

The subclavian vein is one of the most commonly utilized access sites for central venous catheterization. Compared with peripheral venous catheterization, it offers advantages such as longer indwelling duration, capability for rapid fluid resuscitation, and suitability for administering high-concentration or highly irritating medications. It also enables central venous pressure monitoring. Due to its relatively fixed placement, it imposes minimal restrictions on patients’ daily activities. However, the procedure requires high technical proficiency and clinical experience, with precise puncture localization; novice operators experience a considerably higher failure rate. Moreover, blind puncture increases the risk of complications, which is significantly exacerbated in patients with obesity, thoracic deformities, or anatomical variations. Potential complications during or after central venous catheterization include: 1) Postprocedure puncture site pain, local hematoma, and oozing; 2) Puncture site infection; 3) Pneumothorax; 4) Accidental arterial puncture, which may cause life-threatening massive hemorrhage; 5) Gas embolism and other unpredictable life-threatening or disabling events; 6) Failed subclavian vein puncture, necessitating conversion to alternative venous accesses; 7) Catheter occlusion due to coagulation; and 8) Other unforeseen complications, such as respiratory and cardiac arrest due to local anesthetic allergy[1,2].

The patient in this case was critically ill with multiple comorbidities. Compounding these issues were poor patient cooperation and positional limitations, which increased procedural difficulty and led to accidental placement of the left subclavian catheter into the aortic arch. Accidental cannulation of the subclavian artery during subclavian vein catheterization is relatively common, with an incidence of up to 2.7%. In contrast, accidental puncture into the aortic arch is rarely reported in domestic and international literature, leaving little empirical evidence for selecting catheter removal strategies. Additionally, removing the catheter in such cases carries substantial risks, technical challenges, and severe potential complications, including arterial laceration, dissection, pseudoaneurysm, and arteriovenous fistula. Furthermore, catheter surface thrombosis or plaque embolization may cause infarction of vital organs, while mediastinal hematoma can lead to local tissue/organ compression, massive hemorrhage, or even death. These factors necessitate extreme caution when determining the approach for aortic catheter removal. Following a multidisciplinary team consultation and considering the unique aspects of the patient’s condition, we innovatively performed percutaneous aortic occlusion using a VSD occluder, which enabled successful vessel occlusion after catheter removal and minimized the risk of fatal iatrogenic injury [3,4].

## Discussion

This case report describes an instance of accidental aortic arch cannulation in an elderly patient with multiple comorbidities, where the use of a VSD occluder achieved successful catheter removal and hemostasis without subsequent adverse clinical sequelae. As its name indicates, the VSD occluder is primarily indicated for ventricular septal defects, with high success rates and few side effects in managing such congenital heart defects. Its use in the aorta in this case represents an off-label application. While widespread adoption of this technique requires prospective evaluation in large-scale trials, this attempt provides a novel strategy for managing accidental arterial cannulation in noncompressible sites. Additionally, the management of this patient highlights the value of multidisciplinary collaboration, emphasizing optimal disease assessment and treatment selection to better address acute major arterial injuries. Ultimately, we aim to provide a valuable reference with this case report for clinicians encountering similar challenges.

## Patient consent

I have carefully read and fully understood all the above information. The attending physician has provided a detailed explanation of the relevant issues, and I am clearly aware of the risks, benefits, and alternative plans of the proposed diagnosis and treatment plan. Hereby, I voluntarily consent to receive the aforementioned diagnosis and treatment plan and assume the corresponding medical risks.
